# Clinical analysis of 173 pediatric patients with antibody-mediated autoimmune diseases of the central nervous system: a single-center cohort study

**DOI:** 10.3389/fimmu.2023.1140872

**Published:** 2023-04-21

**Authors:** Qingyun Kang, Hongmei Liao, Liming Yang, Hongjun Fang, Zeshu Ning, Caishi Liao, Siyi Gan, Liwen Wu

**Affiliations:** Department of Neurology, Hunan Children’s Hospital, Changsha, China

**Keywords:** antibody-mediated autoimmune diseases, central nervous system, children, clinical features, immunotherapy, prognosis

## Abstract

**Background:**

Antibody-mediated disorders of the central nervous system (CNS) have seen a gradual rise in their incidence and prevalence. This retrospective observational study aimed to investigate the clinical characteristics and short-term prognosis of children with antibody-mediated CNS autoimmune diseases at Hunan Children’s Hospital.

**Methods:**

We collected the clinical data of 173 pediatric patients diagnosed with antibody-mediated CNS autoimmune diseases between June 2014 and June 2021 and analyzed their demographics, clinical features, imaging and laboratory data, treatment, and prognosis.

**Results:**

A total of 187 patients tested positive for anti-neural antibodies and 173 patients were finally diagnosed with antibody-mediated CNS autoimmune diseases after excluding the 14 false-positive cases through clinical phenotypic evaluation and follow-up of treatment outcomes. Of the 173 confirmed patients, 97 (56.06%) were positive for anti-NMDA-receptor antibody, 48 (27.75%) for anti-MOG antibody, 30 (17.34%) for anti-GFAP antibody, 5 (2.89%) for anti-CASPR2 antibody, 3 (1.73%) for anti-AQP4 antibody, 2 (1.16%) for anti-GABABR antibody, and 1 (0.58%) for anti-LGI1antibody. Anti-NMDAR encephalitis was the most commonly seen among the patients, followed by MOG antibody-associated disorders and autoimmune GFAP astrocytopathy. Psycho-behavioral abnormalities, seizures, involuntary movements, and speech disorder were the most common clinical presentations of anti-NMDAR encephalitis, while fever, headache, and disturbance of consciousness or vision were the most seen among patients with MOG antibody-associated disorders or autoimmune GFAP astrocytopathy. The coexistence of multiple anti-neural antibodies was detected in 13 patients, among which 6 cases had coexistent anti-NMDAR and anti-MOG antibodies (including 1 case with anti-GFAP antibody also), 3 cases had coexistent anti-NMDAR and anti-GFAP antibodies, 3 cases had coexistent anti-MOG and anti-GFAP antibodies, 1 case had coexistent anti-NMDAR and anti-CASPR2 antibodies, and 1 case had coexistent anti-GABABR and anti-CASPR2 antibodies. All the survivors were followed up for at least 12 months; 137 recovered completely, 33 had varying sequelae, and 3 died; 22 had one or more relapses.

**Conclusion:**

Antibody-mediated CNS autoimmune diseases occur in children of all ages. Most such pediatric patients have a good response to immunotherapy. Despite the low mortality rate, some survivors have a non-negligible risk of developing relapses.

## 1 Introduction

Autoimmune diseases of the central nervous system (CNS) in children are a group of complex disorders with obvious heterogeneity in pathophysiological mechanisms and clinical manifestations. Antibody-mediated autoimmune diseases represent the most common subgroup. Antibody-mediated CNS disorders represent a distinct subgroup of immune-mediated neurologic disorders characterized by the presence of autoantibodies directed against specific neuronal or glial target antigens mostly expressed in the CNS, which share several distinctive clinical and magnetic resonance imaging (MRI) features (1). Their spectrum ranges from disorders mainly involving white matter such as acquired demyelinating syndromes to disorders mainly involving gray matter such as autoimmune encephalitis. Over the past ten years, antibody-mediated CNS autoimmune diseases have been an important frontier of neuro-immunity and even neurology. Clinical application of anti-neural antibodies has been increasing and new anti-neural antibodies continue to be discovered. The antibody spectrum of CNS autoimmune diseases is also rapidly expanding with the growing understanding of their etiology, pathogenesis, and treatment (2–6).

Antibody-mediated CNS autoimmune diseases will affect patients’ quality of life and bring serious economic burdens to society and their families (7). Most such diseases are sensitive to immunotherapy. Previous studies have emphasized that early diagnosis and timely immunotherapy are the keys to improving the prognosis (8, 9). Early and accurate identification of related antibodies is crucial for the diagnosis of such diseases. Yet, the escalating popularity of such anti-neural antibody tests has increased the number of false-positive cases in clinical practice (10–13). It is of great significance for clinical work to exclude these false-positive cases.

Although there were some cohort studies about autoimmune encephalitis, MOG antibody-associated disorders (MOG-AD), or autoimmune GFAP astrocytopathy (GFAP-A) (14–16), such studies involving pediatric individuals are still limited. So far, there were no cohort studies about multiple subtypes of antibody-mediated CNS autoimmune diseases. Research on the clinical characteristics of antibody-mediated CNS autoimmune diseases, their diagnosis, and their prognostic factors using a large sample size that includes multiple subtypes is warranted. In this work, we retrieved and obtained the data of pediatric patients suspected of having antibody-mediated CNS autoimmune diseases admitted to the Department of Neurology of Hunan Children’s Hospital for detailed analysis. We determined the positive detection rate of anti-neural antibody tests in our hospital and summarized and analyzed the clinical phenotypes, auxiliary examinations, immunotherapy scheme, and short-term prognosis of these patients, to provide a reference for clinical diagnosis and treatment of antibody-mediated CNS autoimmune diseases in children.

## 2 Materials and methods

### 2.1 Participants and samples

Pediatric patients who were suspected of having antibody-mediated CNS autoimmune diseases were collected consecutively from June 2014 to June 2021 at Hunan Children’s Hospital. The included patients in the study were patients who met the diagnostic criteria for probable autoimmune encephalitis (17–19) or acquired demyelinating syndromes (20–22), and patients suspected of having MOG-AD or autoimmune GFAP-A were also enrolled. Their cerebrospinal fluid (CSF) or blood serum tested positive for neural autoantibodies (NMDAR, CASPR2, AMPA1R, AMPA2R, GABABR, LGI-1, MOG, GFAP, AQP4) based on cell-based assays. Patients with alternative causes such as intracranial infections could be reasonably excluded.

The clinical phenotypes of antibody-mediated CNS autoimmune diseases with positive MOG and GFAP antibodies among the included patients were classified by pediatric neurologists. Clinical phenotype such as acute disseminated encephalomyelitis, optic neuritis, transverse myelitis, neuromyelitis optica spectrum disorder, and overlapping syndrome were determined based on the corresponding diagnostic criteria (20, 23, 24). As to meningitis, encephalitis, meningoencephalitis, and encephalomyelitis, the relevant practice recommendations proposed by Hesham Abboud (19) were used for their diagnoses.

A total of 228 serum and 199 CSF samples were collected from 236 pediatric patients suspected of having CNS autoimmune diseases. Autoantibodies against NMDAR, GABABR, LGI1, AMPA1, AMPA2, and CASPR2 were assessed for 116 serum samples and 102 CSF samples. Autoantibodies against AQP4, MBP, MOG, and GFAP were assessed for 48 serum samples and 43 CSF samples. Autoantibodies against NMDAR, GABABR, LGI1, AMPA1, AMPA2, CASPR2, AQP4, MBP, MOG, and GFAP were assessed for 64 serum samples and 54 CSF samples. The blood or CSF samples were sent to Guangzhou Medical Laboratory Center and Kindstar Medical Laboratory (China) for antibody testing. The two laboratories used cell-based assays with high specificity and sensitivity for antibody analysis of the CSF and serum samples. The initial dilution titers of serum and CSF were 1:10 and 1:1, respectively.

### 2.2 Clinical data analysis

The clinical data used for this retrospective analysis included demographic characteristics, clinical manifestations, MRI findings, video electroencephalogram (EEG) data, serum tumor biomarkers, CSF findings, the findings of ultrasound or computed tomography scan of the chest, abdomen, and pelvis cavity, treatment regimens, and prognosis. The follow-up duration was at least 12 months for all included patients. Follow-up visits were carried out every three months during the first year after discharge and every six months thereafter. Modified Rankin Scale (mRS) scoring (for measuring neurological outcomes and assessing the degree of disability) was performed at the onset (initial score), at the time when a patient was in serious condition (maximum score), and at the patient’s last follow-up visit (terminal score). The absence of sequelae represented a good prognosis and otherwise a poor prognosis. The recurrence of multiple subtypes of antibody-mediated CNS autoimmune diseases was defined as the new onset or deterioration of symptoms occurring at least two months after condition improvement or stabilization. The relapses of MOG-AD were defined as the development of new neurological symptoms one month after the initial episode or, in the case of phenotype of acute disseminated encephalomyelitis (ADEM), 3 months after onset of the initial episode (25).

### 2.3 Statistical analysis

All statistical analyses were performed using IBM SPSS 22.0 software. The measurement data were expressed as the mean ± standard deviation (SD). The independent two-sample *t*-test and analysis of variance were performed for multi-group comparisons. SNK-q test was used for pairwise comparison. The paired t-test was for the comparison between the initial mRS score and the terminal mRS score. The enumeration data were expressed as number (n) and percentage (%). The chi-square test or Fisher’s exact test was used for multi-group comparisons. Pairwise comparisons between two groups were adjusted for p-values using the Bonferroni method to retain the nominal alpha value (an adjusted p-value was equal to three times the original p-value). The significance level (α) was set at 0.05, and p-values less than 0.05 were considered statistically significant.

## 3 Results

**Figure 1:**
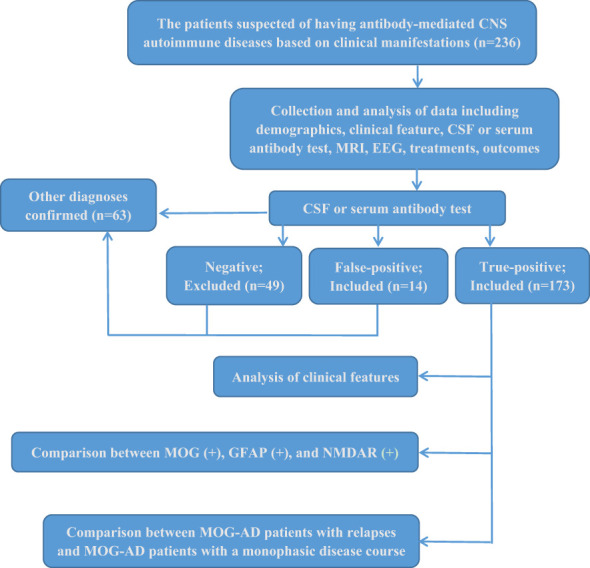
Study flow diagram.

Of the 236 collected patients suspected of having antibody-mediated CNS autoimmune diseases in this study, 187 were positive for anti-neural antibodies, 14 were identified as false-positive cases from further clinical analysis, and 173 defined patients were ultimately enrolled in our further investigation. Only 73.3% of the 236 patients had true positive results for antibody testing. None of the 173 included patients met the revised criteria of the International Pediatric Multiple Sclerosis Study Group (2013) for pediatric multiple sclerosis (20). The flow diagram of this study is shown in **Figure 1**.

### 3.1 False positive antibody test

Based on the clinical manifestations, MRI and EEG findings, CSF changes, antibody titers, response to immunotherapy, and other significant findings (such as past history and pathological examinations), we conducted a comprehensive analysis of the 187 patients positive for anti-neural antibodies to distinguish true-positive from false-positive cases of neural autoantibody-related autoimmunity.

Of the 98 anti-NMDAR-Ab-positive pediatric patients, one patient had positive anti-NMDAR antibody only in serum (see details of patient #1 in **Table 1**). We could not confirm that his etiology was related to the anti-NMDAR antibody. Recurrent seizures were the core manifestation, the antibody titer was low (1:10), and the CSF and MRI results showed no specific changes. The immunotherapy regimen for this patient was ineffective. This patient was finally diagnosed with hereditary epilepsy and excluded from our further study.

**Table 1:** The excluded pediatric patients with false-positive results of anti-neural antibody testing for suspected antibody-mediated CNS autoimmune diseases.

Patient number	Age (years)	Sex	Positive antibody	The serum test result	The cerebrospinal fluid test result	Findings of magnetic resonance imaging	Main clinical manifestations	Possible alternative diagnosis
1	4	Male	NMDAR	Positive (1:10)	Negative	Normal	Seizures	Epilepsy
2	10	Female	MOG	Positive (1:10)	Negative	T2-hyperintense lesions in bilateral frontal lobes and left basal ganglia	Fever, headache, vomiting, lethargy	Cryptococcal neoformans encephalitis
3	8	Male	MOG	Positive (1:10)	Positive (1:10)	T2-hyperintense lesions in bilateral frontal lobes	Seizures	Epilepsy
4	12	Male	MOG	Positive (1:10)	Negative	T2-hyperintense lesions in bilateral frontal lobes and temporal lobe	Weakness, ataxia	Guillain-Barre syndrome
5	4	Female	MOG	Positive (1:10)	Negative	T2-hyperintense lesions in the right temporal lobe	Ataxia, headache	Acute cerebellar ataxia
6	3	Female	MOG	Positive (1:10)	Negative	Normal	Blepharoptosis, disturbance of eye movement	Myasthenia gravis
7	2	Female	GFAP	Positive (1:100)	Negative	Space-occupying lesions in the suprasellar region combined with supratentorial hydrocephalus	Fever, dyskinesia	Brain glioma
8	3	Male	GFAP	Positive (1:100)	Negative	T2-hyperintense lesions in the cerebral peduncle, midbrain, basal ganglia, dorsal thalamus, and temporal lobe	Epilepsy (focal attack)	Brain glioma
9	11	Male	GFAP	Positive (1:100)	Negative	T2-hyperintense lesions in the white matter around the posterior horn of bilateral lateral ventricles	Vertigo	Positional vertigo
10	12	Female	GFAP	Positive (1:32)	Negative	T2-hyperintense lesions in the bilateral subcortical white matter of frontal, parietal, and temporal lobes	Headache	Vascular headache
11	6	Male	GFAP	Positive (1:100)	Negative	T2-hyperintense lesions in the para-Sylvian cistern of the left frontal lobe, and right parietal lobe	Seizures	Epilepsy
12	8	Male	CASPR2	Positive (1:10)	Negative	T2-hyperintense lesions in the white matter around bilateral lateral ventricles	Vomiting, psycho-behavioral abnormalities	Urea cycle disorder
13	6	Male	CASPR2	Positive (1:32)	Unavailable	T2-hyperintense lesions in the bilateral subcortical white matter of the parietal, frontal, and temporal lobes	Seizures	Tuberous sclerosis
14	5	Female	CASPR2	Positive (1:10)	Unavailable	Normal	Seizures	Epilepsy

Of the 53 anti-MOG-Ab-positive patients, five (patients #2–#6; see their details in **Table 1**) were excluded by further check-ups. The fungus *Cryptococcus neoformans* was detected using metagenomic next-generation sequencing of CSF and combined administration of amphotericin B and flucytosine was effective for patient #2, thus this patient was finally diagnosed with cryptococcal meningitis and excluded. The head MRI of patients #3–#5 suggested the possible presence of demyelinating lesions, and several head MRI examinations after immunotherapy did not show any shrinking of the foci, which did not conform to the clinical manifestations and prognosis of MOG-AD. Since the neostigmine test was positive and the administration of pyridostigmine bromide was effective, patient #6 was finally diagnosed with myasthenia gravis and excluded from further analysis.

Of the 35 anti-GFAP-Ab-positive patients, five (patients #7–#11; see details in **Table 1**) were excluded by further check-ups. Patients #7 and #8 were finally diagnosed with glioma by brain biopsy. Among patients #9–#11, no solid evidence of encephalopathy or encephalitis, and no specific changes in CSF were found; their intracranial lesions did not shrink after immunotherapy, and the use of immunotherapy had little effect on their prognosis. These five patients were not considered to have autoimmune GFAP-A and were excluded from our further analysis.

Besides, three patients (patients #12–#14; see their details in **Table 1**), of the eight anti-CASPR2-Ab-positive patients, were excluded from our further analysis. In detail, patient #12 was diagnosed with tuberous sclerosis due to multiple Hypomelanotic macules throughout the body, multiple calcified subependymal nodules revealed by head CT, and a novel TSC1 pathogenic variant revealed by whole exome sequencing. Patient #13 was diagnosed with a urea cycle disorder due to abnormally elevated blood ammonia level, significantly increased citrulline concentration in dried blood spots revealed by tandem mass spectrometry, elevated orotic acid level in the urine, and ASSI pathogenic variants revealed by whole exome sequencing. Patient #14 was diagnosed with hereditary epilepsy; recurrent seizures were the core manifestation, the antibody titer was low (1:10), and the MRI results showed no specific changes; the immunotherapy regimen for this patient was ineffective.

### 3.2 Antibody positivity rate

Of the 97 anti-NMDAR-Ab-positive encephalitis patients, 92 received testing for anti-NMDAR antibodies in CSF, and 88 were positive (95.65%); 92 received testing for anti-NMDAR antibodies in serum, and 78 were positive (84.78%). Of the 48 patients with MOG-AD, 47 received testing for anti-MOG antibodies in serum, and all were positive (100%); 38 received testing for anti-MOG antibodies in CSF, and 21 were positive (43.75%). Of the 30 autoimmune GFAP-A patients, 29 received testing for anti-GFAP antibodies in serum, and 25 were positive (86.21%); 24 received testing for anti-GFAP antibodies in CSF, and 15 were positive (62.5%). The five anti-CASPR2-Ab-positive encephalitis patients received testing for anti-CASPR2 antibodies in serum and CSF, five were positive for the antibodies in serum (100%) and two were positive for the antibodies in CSF (40%). Of the two anti-GABABR-Ab-positive encephalitis patients, two were positive for the antibody in serum (100%) and one was positive for the antibody in CSF (50%). Besides, one anti-LGI1-Ab-positive encephalitis patient was positive for the antibody in serum and CSF (100%).

### 3.3 Coexistence of antibodies

In this cohort study, many patients had coexistent multiple anti-neural antibodies, and the coexistence of anti-MOG antibody and anti-NMDAR antibody was the most common. A total of 13 patients with coexistent multiple anti-neural antibodies were found in our included cases. In detail, six patients had coexistent anti-NMDAR antibody and anti-MOG antibody (including one with GFAP antibody), three had coexistent anti-NMDAR antibody and anti-GFAP antibody, three had coexistent anti-MOG antibody and anti-GFAP antibody, one patient had coexistent anti-NMDAR antibody and anti-CASPR2 antibody, and one patient had coexistent anti-GABABR antibody and anti-CASPR2 antibody. The coexistence of anti-neural antibodies is not uncommon for patients with Systemic lupus erythematous, but none of patients had co-existing Systemic lupus erythematous in our study.

### 3.4 Clinical features

**Figure 2:**
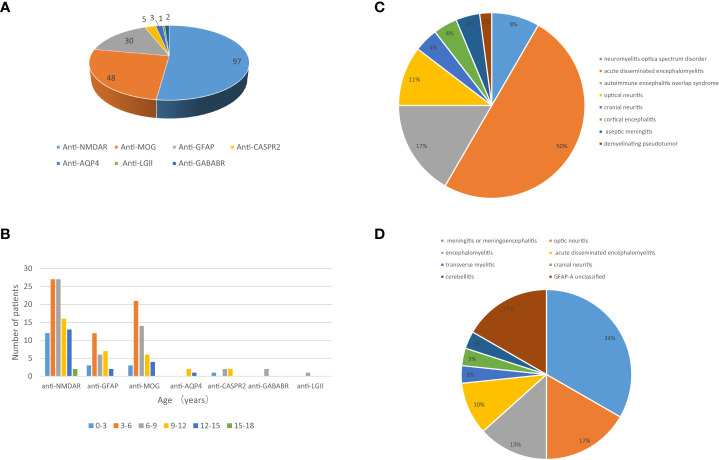
The profile of the included patients with antibody-mediated CNS autoimmune diseases. **(A)** Subtypes of antibody-mediated CNS autoimmune diseases in pediatric patients and **(B)** age distribution of these patients. The clinical phenotypes of 48 pediatric patients with MOG-AD **(C)** and 30 pediatric patients with autoimmune GFAP-A **(D)**.

A total of 173 patients were finally diagnosed with antibody-mediated CNS autoimmune diseases after excluding the 14 false-positive cases. Their detailed clinical data were collected for analysis. Anti-NMDAR encephalitis was the most common subtype (97 cases), followed by MOG-AD (48 cases) and autoimmune GFAP-A (30 cases) (**Figure 2A**). Five or fewer patients with antibody-mediated CNS autoimmune diseases had anti-CASPR2, anti-AQP4, anti-GABABR, or anti-LGI1 antibodies (**Figure 2A**). Of the 173 patients, 82 were male and 91 were female; the oldest was 16 years old, and the youngest patient was only 3 months old, and the peak ages of onset were between 3 and 6 years and between 6 and 9 years (**Figure 2B**). The demographics of the included patients were shown in **Table 2**.

**Table 2:** Demographics, clinical features, auxiliary examinations, treatments, and outcomes of pediatric patients with antibody-mediated autoimmune diseases of the central nervous system.

	Pediatric patients							
	Total (n=173)	MOG (n=48)	GFAP (n=30)	AQP4 (n=3)	NMDAR (n=97)	CASPR2 (n=5)	GABABR (n=2)	LGI1 (n=1)
Demographics								
Range of onset age (months)	3–198	13–166	14–154	108–179	3–192	25–118	88–118	157
Mean onset age (years)	7.06 ± 3.6	6.57 ± 3.02	6.77 ± 3.34	11.81 ± 2.97	7.16 ± 3,79	2.95 ± 1.63	4.25 ± 1.89	_
Sex (male/female)	82/91	18/30	19/11	0/3	42/55	4/1	2/0	0/1
Types of onsets (n)								
Acute (≤2 weeks)	106	31	19	3	60	2	1	1
Sub-acute (2 weeks ~ 1 month)	42	11	8	0	21	3	1	0
Chronic (≥1 month)	25	6	3	0	16	0	0	0
Initial manifestations (n)								
Seizures	59	6	5	0	50	2	1	0
Psycho-behavioral abnormalities	26	0	1	0	22	2	1	1
Fever	33	15	13	0	6	0	0	0
Visual disturbance	14	11	3	1	1	0	0	0
Others	41	16	8	2	18	1	0	0
Clinical manifestations (n)								
Seizures	88	14	11	0	64	3	2	1
Status epilepticus	33	4	2	0	25	1	1	1
Consciousness disturbance	92	26	15	0	51	3	2	1
Fever	82	28	17	0	39	1	2	1
Psycho-behavioral abnormalities	103	10	12	0	81	5	1	1
Headache	64	20	13	1	32	0	0	1
Memory deficit	60	10	6	2	36	2	2	1
Speech disorder	79	12	6	0	61	2	1	1
Sleep disorder	60	3	2	0	53	4	1	1
Involuntary movement	84	4	6	0	65	1	1	0
Visual disturbance	32	18	5	2	9	0	0	0
Ataxia	37	13	7	1	18	0	0	0
Autonomic dysfunction	48	9	9	2	25	3	1	0
Sensory disorder	19	4	5	2	9	0	0	0
Faciobrachial dystonic seizures	1	0	0	0	0	0	0	1
Positive antibody (n, %)								
Only serum	49	26	15	0	9 (9.3%)	3 (60%)	1 (50%)	–
Only cerebrospinal fluid	22	1	5	0	19 (19.6%)	–	–	–
Cerebrospinal fluid and serum	102	21	10	3	69 (71.1%)	2 (40%)	1 (50%)	1 (100%)
Cerebrospinal fluid analysis								
Number of cases with increased protein level (range, mg/L)	25	9 (200–1638)	7 (180–1610)	0	6 (160–860)	1 (593)	0	0
Number of cases with cerebrospinal fluid pleocytosis (>5/mm^3^) (range, 10^6^ cells/L)	133	42 (2–430)	25 (1–600)	2 (4–28)	69 (1–144)	4 (6–214)	2 (15–40)	1 (23)
Cases with abnormalities in brain magnetic resonance imaging (n)								
Total	105	42	25	1	42	2	2	1
Frontal lobe	66	33	16	0	25	1	1	0
Parietal lobe	64	27	14	0	28	1	1	0
Temporal lobe	53	21	9	0	21	1	1	1
Occipital lobe	44	20	11	0	15	1	1	0
Basal ganglia	28	17	8	0	7	0	1	0
Thalamus	42	16	13	0	15	2	2	0
Brain stem	23	15	10	1	2	0	0	0
Cerebellum	22	15	5	0	6	0	0	0
Callosum	12	8	4	0	3	0	0	0
Insular lobe	14	5	1	0	9	1	1	0
Others	9	3	1	0	2	1	1	0
Cases with abnormalities in spinal cord magnetic resonance imaging (n)								
Cervical cord	–	16	5	3	–	–	–	–
Thoracic cord	–	18	4	1	–	–	–	–
Lumbar cord	–	5	2	0	–	–	–	–
Medullary cone	–	1	0	0	–	–	–	–
Cases with abnormal electroencephalogram (n)								
Total	150	34	21	2	90	5	2	1
Epileptiform discharges	49	4	4	0	39	2	2	1
Slow waves	143	34	20	2	88	5	2	1
Others (n)								
Secondary to herpes simplex encephalitis	6	2	0	0	4	0	0	0
Complicated with tumor(s)	4	0	1	0	2	0	1	0
Treatments (n)								
No immunotherapy	2	1	1	0	0	0	0	0
Only IVIG	6	2	2	0	2	0	0	0
Only steroids	12	9	5	0	0	0	0	0
Steroids + IVIG	153	36	22	3	95	5	2	1
Steroids + IVIG + plasma exchange	5	1	1	0	3	0	0	0
Second-line treatment	33	6	4	2	20	1	1	0
Admission to an intensive care unit	37	4	6	0	26	2	2	0
Mean time to discharge (days)	29.35 ± 20.45	19.92 ± 10.91	24.37 ± 12.69	30.67 ± 12.64	33.77 ± 23.34	35.4 ± 19.5	51 ± 22.63	36
Outcomes								
Complete recovery	137	40	21	1	73	4	1	1
Movement disorder	12	3	3	1	5	0	0	0
Cognitive disorder	26	5	5	1	15	1	0	0
Epilepsy	13	4	2	0	7	0	0	0
Visual disturbance	2	1	0	1	0	0	0	0
Relapses	22	11	5	2	8	0	0	0
Mortality	3	0	0	0	2	0	1	0
Average maximum mRS score	3.65 ± 1.02	3.5 ± 0.88	3.47 ± 1.25	3.33 ± 0.58	3.67 ± 1.01	3.8 ± 1.3	5 ± 0	4
Average terminal mRS score	0.6 ± 1.26	0.25 ± 0.67	0.8 ± 1.47	1.67 ± 1.53	0.59 ± 1.23	0.2 ± 0.45	3 ± 4.24	0

NMDAR, N-methyl-D-aspartate receptor; AQP4, aquaporin 4; CASPR2, contactin-associated protein-like 2; MOG, myelin oligodendrocyte glycoprotein; GABABR, γ-aminobutyric acid B receptor; GFAP, glial fibrillary acidic protein; LGI1, leucine-rich glioma-inactivated 1; IVIG, intravenous immunoglobulin; mRS, modified Rankin scale.

Of the 173 included patients, 106 (61.27%) had acute onset (≤2 weeks), 42 (24.27%) had subacute onset (2 weeks ~ 1 month), and 25 (14.45%) had chronic onset (≥1 month). Among anti-NMDAR encephalitis patients, psycho-behavioral abnormalities, seizures, involuntary movements, and speech disorder were the most common clinical manifestations; other common symptoms included consciousness disturbance, memory loss, sleep disorder, headache, fever, and autonomic nervous dysfunction; paresthesia, ataxia, and visual disturbance were also found. Among the patients with MOG antibody associated-disorders, fever (the most common), consciousness disturbance, visual disturbance, headache, and seizures were the common clinical manifestations. Of the 48 cases with MOG-AD, 24 had acute disseminated encephalomyelitis, 8 had autoimmune encephalitis overlap syndrome, 5 had optic neuritis, 4 had neuromyelitis optica spectrum disorder, and the resting cases had cortical encephalitis, aseptic meningitis, cranial neuritis, or demyelinating pseudotumor (**Figure 2C**). Autoimmune GFAP-A patients commonly had headaches, fevers, consciousness disturbance, and seizures. Of the 30 autoimmune GFAP-A patients, 10 had meningitis or meningoencephalitis, 4 had encephalomyelitis, 5 had optic neuritis, 3 had acute disseminated encephalomyelitis, and the rest had transverse myelitis, cranial neuritis, or cerebellitis (**Figure 2D**). Hyponatremia and faciobrachial dystonic seizures were seen among the anti-LGI1 encephalitis patients (**Table 2**).

### 3.5 Auxiliary examinations

Of the 173 included cases, 168 received CSF examination; 133 had leukocytosis (leukocyte count > 5 cells/mm^3^), with the highest record of 600 cells/mm^3^, and 25 had an elevated protein level (> 500 mg/L), with the highest record of 1,638 mg/L. The oligoclonal bands were detected in 107 patients, and 21 positive results were obtained. The anti-nuclear antibody (ANA) and anti-double-stranded DNA (anti-dsDNA) antibody were tested in 103 patients, but there were no positive findings. Besides, the elevation of adenosine deaminase (ADA) in CSF was detected in 5 of 30 autoimmune GFAP-A patients. All our included patients received a head MRI. The distribution of lesions was displayed in **Table 2**. EEG abnormalities were observed in 150 of 169 patients receiving EEG. Most abnormal cases exhibited local or global slow wave activity and some showed the characteristics of epileptiform discharges in EEG. The included patients were screened for potential tumors; three had ovarian teratoma and one had neuroblastoma. The detection of HSV PCR or HSV IgM in CSF indicated that four patients with anti-NMDAR encephalitis and two patients with MOG-AD were secondary to herpes simplex encephalitis (**Table 2**).

### 3.6 Treatment and follow-up

In our cohort, patients with antibody-mediated CNS autoimmune diseases, especially those with anti-NMDAR encephalitis, generally had a long hospital stay (**Table 2**). A total of 171 patients (98.84%) received immunotherapy. Immunotherapy included first-line (intravenous immunoglobulins, glucocorticoids, and plasma exchange) and second-line (rituximab, cyclophosphamide, and azathioprine) therapies. The different immunotherapies used in each subgroup of antibody-mediated CNS autoimmune diseases were shown in **Table 2**. All 97 anti-NMDAR encephalitis patients received first-line immunotherapy and 95 of them received combination therapy. Twenty anti-NMDAR encephalitis patients who responded poorly to first-line treatments or experienced relapse(s) received second-line immunotherapy; 19 were treated with rituximab and one was treated with cyclophosphamide. Besides, two anti-NMDAR encephalitis patients received surgical removal of their ovarian teratoma. Of the 48 patients with MOG-AD, 47 received first-line immunotherapy and 36 of them received combination therapy, and one did not receive immunotherapy due to spontaneous remission of symptoms. Six patients with MOG-AD who responded poorly to first-line treatments or experienced relapse(s) received second-line immunotherapy; five were treated with rituximab and one was treated with azathioprine. Of the 30 autoimmune GFAP-A patients, 29 received first-line immunotherapy and 22 of them received combination therapy, and one patient did not receive immunotherapy because of spontaneous remission of symptoms. Of the four recurrent patients, three were treated with rituximab and one was treated with azathioprine.

All included patients were followed up for at least 12 months after discharge. Of the 97 patients with anti-NMDAR encephalitis, 73 (75.3%) recovered completely, two (2.1%) died, and 22 (22.7%) had varying degrees of sequelae; eight patients (8.2%) experienced relapse(s) during the follow-up. Of the 30 patients with autoimmune GFAP-A, 21 recovered completely, 9 had varying degrees of sequelae, and 5 experienced relapse(s). Of the 48 patients with MOG-AD, 40 recovered completely, and eight had varying degrees of sequelae; 11 patients relapsed during the follow-up. As shown in **Table 3**, the MOG-AD patients with relapse(s) had fewer spinal MRI abnormalities,less consciousness disturbance and less fever but more visual disturbance than those with a monophasic disease course (p<0.05).

**Table 3:** Comparison between MOG-AD patients with relapses and such patients with a monophasic disease course.

Variable	Total	Patients with relapse(s) (n=11)	Patients with a monophasic disease course (n=37)	t/χ2	p
Sex, male	29 (60.42%)	6 (54.55%)	23 (62.16%)	*	0.732
Age	78.88 ± 36.26	96 ± 39.14	73.78 ± 34.28	1.828	0.074
Length of stay	19.92 ± 10.91	17.64 ± 5.32	20.59 ± 12.06	−1.16	0.253
Initial mRS score	3.5 ± 0.88	3.27 ± 1.01	3.57 ± 0.83	−0.981	0.332
Terminal mRS score	0.25 ± 0.67	0.18 ± 0.4^a^	0.27 ± 0.73^a^	−0.516	0.609
Leukocytosis	42 (87.50%)	10 (90.91%)	32 (86.49%)	*	>0.999
Abnormal head MRI scan	42 (87.50%)	9 (81.82%)	33 (89.19%)	*	0.609
Abnormal orbit MRI scan	16 (33.33%)	5 (45.45%)	11 (29.73%)	*	0.468
Abnormal spinal MRI scan	19 (39.58%)	1 (9.09%)	18 (48.65%)	*	0.032
Abnormal electroencephalogram	34 (85.00)	8 (80.00%)^b^	26 (86.67%)^c^	*	0.629
Fever	28 (58.33%)	3 (27.27%)	25 (67.57%)	*	0.034
Seizure(s)	14 (29.17%)	5 (45.45%)	9 (24.32%)	*	0.258
Status epilepticus	4 (8.33%)	2 (18.18%)	2 (5.41%)	*	0.221
Psycho-behavioral abnormalities	10 (20.83%)	3 (27.27%)	7 (18.92%)	*	0.675
Consciousness disturbance	26 (54.17%)	2 (18.18%)	24 (64.86%)	7.443	0.006
Visual disturbance	18 (47.37%)	8 (72.73%)	10 (37.04%)	3.993	0.046
Positive tests of multiple antibodies	8 (16.67%)	4 (36.36%)	4 (10.81%)	*	0.068
Antibody titer ≥1:100	26 (54.17%)	7 (63.64%)	19 (51.35%)	0.515	0.473

The asterisks (*) indicate no statistics for Fisher’s exact test. P^a^ < 0.05 compared with the initial mRS score. One^b^ and seven^c^ patients did not receive an electroencephalogram test. MRI, magnetic resonance imaging; mRS, modified Rankin scale.

### 3.7 Comparisons between pediatric patients with anti-NMDAR encephalitis, MOG-AD, and autoimmune GFAP-A

In this study, there were 97 patients with anti-NMDAR encephalitis, 48 MOG-AD patients, and 30 patients with autoimmune GFAP-A. Among the patients with anti-NMDAR encephalitis, psycho-behavioral abnormalities (the most common), seizures, involuntary movements, consciousness disturbance, speech disorder, cognitive disorder, sleep disorder, and autonomic dysfunction were the common clinical manifestations. Among the MOG-AD and autoimmune GFAP-A patients, fever (the most common), consciousness disturbance, visual disturbance, headache, and seizures were the common manifestations. Between the MOG-AD and autoimmune GFAP-A patients, no significant difference was seen in the incidence of clinical manifestations including fever, seizures, status epilepticus, psycho-behavioral abnormalities, consciousness disturbance, and visual disturbance, and in the length of hospital stay, abnormality rates of head MRI and EEG, complete recovery rate, and recurrence rate. The anti-NMDAR encephalitis patients had longer hospital and higher incidence of clinical manifestations including psycho-behavioral abnormalities, involuntary movements, speech disorder, and sleep disorder than the MOG-AD patients and autoimmune GFAP-A patients. In addition, the incidences of seizures, status epilepticus, and admission to the intensive care unit in the anti-NMDAR encephalitis patients were significantly higher than those in the MOG-AD patients (p<0.05), while the incidence of visual disturbance in the anti-NMDAR encephalitis patients was significantly lower than those in the MOG-AD patients (p<0.05). Compared with MOG-AD patients, anti-NMDAR encephalitis patients had a higher abnormal EEG rate and a lower abnormal head MRI rate. Although the anti-NMDAR encephalitis patients usually required longer hospital stays, they showed no significant difference in the complete recovery rate and recurrence rate from the MOG-AD or autoimmune GFAP-A patients (p<0.05). More details are shown in **Table 4**.

**Table 4:** Comparisons among the MOG group, GFAP group, and NMDAR group.

Variables	Total	MOG (n=48)	GFAP (n=30)	NMDAR (n=97)	t/χ2	p
Time to discharge (days)	28.36 ± 19.96	19.92 ± 10.91	24.37 ± 12.69	33.77 ± 23.34^ab^	9.269	<0.001
Average maximum mRS score	3.59 ± 1.02	3.5 ± 0.88	3.47 ± 1.25	3.67 ± 1.01	0.705	0.495
Average terminal mRS score	0.53 ± 1.16	0.25 ± 0.67	0.8 ± 1.47	0.59 ± 1.23	2.352	0.098
Onset age (months)	83.73 ± 41.57	78.88 ± 36.26	81.2 ± 40.08	86.91 ± 44.49	0.664	0.516
Complete recovery	134 (63.21%)	40 (63.49%)	21 (65.63%)	73 (62.39%)	0.116	0.944
Recurrence	24 (11.32%)	11 (17.46%)	5 (15.63%)	8 (6.84%)	5.298	0.071
Abnormal head MRI scan	109 (51.42%)	42 (66.67%)	25(78.13%)	42 (35.90%)^ab^	26.284	<0.001
Abnormal electroencephalogram	145 (68.40%)	34 (53.97%)	21 (65.63%)	90 (76.92%)^a^	10.116	0.006
Abnormal leukocyte count in CSF	136 (64.15%)	42 (66.67%)	25 (78.13%)	69 (58.97%)	4.254	0.119
Fever	84 (39.62%)	28 (44.44%)	17 (53.13%)	39 (33.33%)	4.985	0.083
Seizures	89 (41.98%)	14 (22.22%)	11 (34.38%)	64 (54.70%)^a^	18.630	<0.001
Status epilepticus	31 (14.62%)	4 (6.35%)	2 (6.25%)	25 (21.37%)^a^	9.514	0.009
Psycho-behavioral abnormalities	103 (48.58%)	10 (15.87%)	12 (37.50%)	81 (69.23%)^ab^	48.526	<0.001
Consciousness disturbance	92 (43.40%)	26 (41.27%)	15 (46.88%)	51 (43.59%)	0.275	0.871
Visual disturbance	32 (15.09%)	18 (28.57%)	5 (15.63%)	9 (7.69%)^a^	13.938	0.001
Involuntary movements	75 (35.38%)	4 (6.35%)	6 (18.75%)	65 (55.56%)^ab^	47.927	<0.001
Ataxia	38 (17.92%)	13 (20.63%)	7 (21.88%)	18 (15.38%)	1.167	0.558
Speech disorder	79 (37.26%)	12 (19.05%)	6 (18.75%)	61 (52.14%)^ab^	24.705	<0.001
Sleep disorder	58 (27.36%)	3 (4.76%)	2 (6.25%)	53 (45.30%)^ab^	42.310	<0.001
Admission to an intensive care unit	36 (16.98%)	4 (6.35%)	6 (18.75%)	26 (22.22%)^a^	7.402	0.025

a p<0.05, compared with MOG group; ^b^p<0.05, compared with GFAP group.

## 4 Discussion

A spectrum of patients with neurological and psychiatric symptoms have been diagnosed with antibody-mediated CNS autoimmune diseases with the increasing awareness of such diseases and unceasing discovery of related autoantibodies. However, relatively few studies have focused on pediatric patients with such autoimmune diseases (26–28). This retrospective, observational, single-center study collected the clinical data of 173 pediatric patients diagnosed with antibody-mediated CNS autoimmune diseases and analyzed their demographics, clinical features, laboratory and imaging data, treatment, and prognosis.

Of our 173 subjects, 97 were patients with anti-NMDAR encephalitis, similar to previous reports (29). Anti-NMDAR encephalitis is the most common type of autoimmune encephalitis in children, and its clinical diagnosis is not difficult because most of its pediatric patients have typical clinical manifestations. The detection of anti-NMDAR antibodies should be administered to patients with acute- or subacute-onset repeated seizures, unexplained psycho-behavioral abnormalities, or involuntary movements.

Over 90% of the early reported pediatric cases with MOG-AD had classical acquired demyelinating syndromes of the CNS such as acute disseminated encephalomyelitis, optic neuritis, transverse myelitis, and neuromyelitis optica spectrum disorder (24). In recent years, the clinical phenotype spectrum of this disease is expanding with the deepening understanding of the clinical manifestations, pathophysiology, and pathogenesis of MOG-AD, along with increasing reports of special phenotypes such as cortical encephalitis, demyelinating pseudotumor, cranial neuritis, and aseptic meningitis. Our cohort study included 48 such pediatric patients and acute disseminated encephalomyelitis was found to be the most common clinical phenotype, which was consistent with previous reports (24, 30). However, up to 31% of our confirmed clinical phenotypes did not conform to the diagnostic features of classical demyelinating syndromes of the CNS, which is higher than the earlier reported at home and abroad (31, 32). This may be explained by the increased atypical cases confirmed by the cell-based assays adopted in this study and the increasing MOG antibody tests in children with unexplained encephalitis and white matter lesions. The clinical manifestations of pediatric MOG-AD are highly heterogeneous. In this study, two patients with aseptic encephalitis had repeated fever, headache, and lethargy, and increased white blood cell counts in CSF. No abnormalities were found in their several head MRI scans, and their symptoms were not improved after anti-infection treatment. However, with the serum MOG-Ab-positive results, their symptoms were completely relieved by intravenous immunoglobulins and methylprednisolone pulse therapy. It is suggested that MOG antibody screening for pediatric patients with prolonged fever, lethargy, and leukocytosis in CSF and without sufficient etiological evidence for intracranial infection is needed to avoid missed diagnoses of MOG-AD. Two of the 48 MOG-AD patients were secondary to herpes simplex encephalitis. These two patients developed apathy, seizures, and memory loss during the recovery period; a reexamination of the head MRI revealed new multifocal white matter lesions, and serum MOG antibodies were detected. Thus, they were attributed to MOG-AD cases. It has been previously reported that autoimmune encephalitis secondary to herpes simplex virus encephalitis is mostly anti-NMDAR encephalitis. Whether anti-MOG antibodies could be identified as “responsible antibodies” in these two patients remains to be investigated in further studies.

MOG-AD patients had the highest recurrence rate in our study. How to identify recurrence risk in pediatric individuals with MOG-AD, and effective treatment approaches to preventing recurrence, are hot issues for clinicians. There are few reports with a large sample size and long-term follow-up on MOG-AD recurrence factor analysis in children. Our study showed that MOG-AD patients with relapses had a lower incidence of abnormal spinal MRI,fever and consciousness disturbance and a higher incidence of visual disturbance than the MOG-AD patients with a monophasic disease course. Practitioners need to be alert to the possible recurrence among MOG-AD patients with this manifestation. The common clinical manifestations in the MOG-AD and autoimmune GFAP-A groups were fever, consciousness disturbance, seizures, visual disturbance, speech disorder, headache and ataxia. No significant difference was seen in the incidence of common clinical manifestations, the length of hospital stays, abnormality rates of MRI and EEG, complete recovery rate, and recurrence rate between these two groups. Clinicians must be vigilant to the possible presence of MOG-AD or autoimmune GFAP-A in children when they have a fever of unknown origin, consciousness disturbance, seizures, or visual disturbance and timely performed MOG or GFAP antibody screening to avoid their missed diagnoses. In addition, 5 (16.6%) of our 30 autoimmune GFAP-A patients had; therefore, the possibility of autoimmune GFAP-A, in addition to tuberculous meningitis, should be taken into consideration by the clinicians when in the presence of elevated CSF adenosine deaminase.

The widespread application of anti-neural antibody detection has increased the number of pediatric cases of antibody-mediated CNS autoimmune diseases. However, an antibody positivity test is not always enough to ensure a correct diagnosis of such autoimmune diseases (1). Despite the high specificity of cell-based assays for anti-neural antibody tests, false positive results may occur, especially when antibody titers are low (13, 33). In our research, 9.4% of anti-MOG-Ab-positive cases, 37.5% of anti-CASPR2-Ab-positive cases, and 14.3% of anti-GFAP-Ab-positive cases were confirmed false positives, which is generally consistent with previous reports. False positives of anti-neural antibody tests may occur in pediatric patients with various surrogate nervous system diseases (*e.g.*, infectious, hereditary, neoplastic, metabolic, vascular), but it seems rare in cases without known neurological diseases. This may indicate that given the presence of epitope spreading, the production of anti-neural antibodies may increase due to nervous system damage caused by other causes or cross-reactivity with autoantibodies against alternative antigenic targets.

The coexistence of multiple anti-neural antibodies is found in our cohort study, which is clinically referred to as the presence of coexistent multiple autoantibodies in the same patient at the same time point or different autoantibodies in the same patient at different time points. In our study, 13 patients had coexistent multiple anti-neural autoantibodies, and the coexistence of anti-NMDAR antibody and anti-MOG antibody was the most common type; the pediatric patients with such coexistent antibodies would face a higher incidence of psycho-behavioral abnormalities and frequent seizures than the patients with positive MOG antibody alone, and would more experience demyelinating symptoms such as visual disturbance or have a higher incidence of abnormal head MRI than the patients with positive anti-NMDAR antibody alone, which was consistent with previous reports (34, 35). Besides, the coexistence of anti-NMDAR antibody and anti-GFAP antibody and anti-GFAP antibody and anti-MOG antibody was also common among our cohort cases. In our cohort, all patients suspected of having MOG-AD were tested for anti-AQP4 antibodies, but no patients were double positive for anti-AQP4 and anti-MOG antibodies. To date, the pathological immune mechanisms behind the coexistence of multiple antibodies are still unclear, which may be related to immune reconstruction during viral infection, disease development, and treatment. Given the presence of several kinds of anti-neural antibodies in patients, we should deeply investigate the causal relationship between the detected antibodies and the clinical core phenotype, and clarify the pathogenic antibodies responsible for the causality in the individual disease course. Thus, one or more responsible/pathogenic antibodies should be confirmed based on the clinical core phenotype among the patients with coexistent multiple autoantibodies, to avoid imprudent diagnosis only according to identified antibodies. Besides, the presence of false positives of anti-neural antibody tests and coexistent multiple anti-neural antibodies underscores the importance of correct phenotypic evaluation and follow-up of treatment outcomes. Clinically, except for antibody-positive results, clinical manifestations, disease evolution, findings of imaging and other auxiliary examinations, and even nerve biopsy findings should be combined for comprehensive judgment to achieve an accurate diagnosis and administer reasonable treatments. Beyond that, the deepening understanding of antibody-mediated CNS autoimmune diseases is bringing more challenges to clinical practice.

In conclusion, our cohort study summarized the clinical features and evaluated clinical immunotherapies and short-term prognosis of antibody-mediated CNS autoimmune diseases in children. To some degree, the limited sample size and potential selection bias in this retrospective study limit the extrapolation from our current clinical findings to a wide application in future clinical practice. This cohort study is one of the largest case series studies on antibody-mediated CNS autoimmune diseases among Chinese pediatric patients to date and was conducted in a central medical institution in China, which is representative and could provide a basis for future research. Future prospective multi-center studies with larger sample sizes are warranted to verify our present findings.

## Data availability statement

The original contributions presented in the study are included in the article/supplementary material. Further inquiries can be directed to the corresponding author.

## Ethics statement

The studies involving human participants were reviewed and approved by the Ethics Committee of Hunan Children’s Hospital (Hunan, China). Written informed consent to participate in this study was provided by the participants’ legal guardian/next of kin.

## Author contributions

QK conducted the literature review and drafted the manuscript. HL, LY, HF, ZN, CL, and SG made substantial contributions to the conception and interpretation of data. LW was responsible for revising the manuscript critically and has given final approval for publishing the version. All authors contributed to the article and approved the submitted version.
